# Interplay between intergrin-linked kinase and ribonuclease inhibitor affects growth and metastasis of bladder cancer through signaling ILK pathways

**DOI:** 10.1186/s13046-016-0408-x

**Published:** 2016-08-30

**Authors:** Xiang Zhuang, Mengxin Lv, Zhenyu Zhong, Luyu Zhang, Rong Jiang, Junxia Chen

**Affiliations:** 1 Department of Cell Biology and Genetics, Chongqing Medical University, Chongqing, 400016 China; 2 The First Clinical College, Chongqing Medical University, Chongqing, 400016 China; 3 Molecular Medicine and Cancer Research Center, Chongqing Medical University, Chongqing, 400016 China; 4 Laboratory of Stem Cells and Tissue Engineering, Chongqing Medical University, Chongqing, China

**Keywords:** Interplay, Integrin-linked kinase, Ribonuclease inhibitor, ILK pathway, Epithelial-mesenchymal transition, Bladder cancer cells

## Abstract

**Background:**

Integrin-linked kinase (ILK) is a multifunctional adaptor protein which is involved with protein signalling within cells to modulate malignant (cancer) cell movement, cell cycle, metastasis and epithelial–mesenchymal transition (EMT). Our previous experiment demonstrated that ILK siRNA inhibited the growth and induced apoptosis of bladder cancer cells as well as increased the expression of Ribonuclease inhibitor (RI), an important cytoplasmic protein with many functions. We also reported that RI overexpression inhibited ILK and phosphorylation of AKT and GSK3β. ILK and RI gene both locate on chromosome 11p15 and the two genes are always at the adjacent position of same chromosome during evolution, which suggest that ILK and RI could have some relationship. However, underlying interacting mechanisms remain unclear between them. Here, we postulate that RI might regulate ILK signaling pathway via interacting with ILK.

**Methods:**

Co-immunoprecipitation, GST pull-down and co-localization under laser confocal microscope assay were used to determine the interaction between ILK and RI exogenously and endogenously. Furthermore, we further verified that there is a direct binding between the two proteins by fluorescence resonance energy transfer (FRET) in cells. Next, The effects of interplay between ILK and RI on the key target protein expressions of PI3K/AKT/mTOR signaling pathway were determined by western blot, immunohistochemistry and immunofluorescence assay in vivo and in vitro. Finally, the interaction was assessed using nude mice xenograft model.

**Results:**

We first found that ILK could combine with RI both in vivo and in vitro by GST pull-down, co-immunoprecipitation (Co-IP) and FRET. The protein levels of ILK and RI revealed a significant inverse correlation in vivo and in vitro. Subsequently, The results showed that up-regulating ILK could increase cell proliferation, change cell morphology and regulate cell cycle. We also demonstrated that the overexpression of ILK remarkably promoted EMT and expressions of target molecules of ILK signaling pathways in vitro and in vivo. Finally, we found that ILK overexpression significantly enhanced growth, metastasis and angiogenesis of xenograft tumor; Whereas, RI has a contrary role compared to ILK in vivo and in vitro.

**Conclusions:**

Our findings, for the first time, directly proved that the interplay between ILK and RI regulated EMT via ILK/PI3K/AKT signaling pathways for bladder cancer, which highlights the possibilities that ILK/RI could be valuable markers together for the therapy and diagnosis of human carcinoma of urinary bladder.

## Background

Integrin-linked kinase (ILK), a 59 kDa serine/threonine kinase, was discovered by detecting its interactions with the β1 integrin subunit [1]. ILK regulates a plethora of biological processes such as cell cycle, growth and angiogenesis; high expression of ILK enhances tumorigenesis [2, 3]. ILK directly phosphorylates AKT and GSK-3β to mediate β-catenin for oncogenesis [4]. Over-expression of ILK has a close relationship with prostate cancer, high-grade gastric cancer, and NSCLC (non-small cell lung cancer) [5–7]. In addition, it has been reported that increased expression of the protein is inversely related to the 5-year survival rate in prostate cancer [6]. ILK has important functions in promoting epithelial to mesenchymal transition (EMT) in mammary epithelium and the tumor invasion with a lower expression of E-cadherin [8, 9]. Using a selective inhibitor of ILK has been demonstrated to induce E-cadherin expression [10]. This is supposed to be an effective way to inhibit EMT [11]. However, the molecular basis for the high level expression and regulatory mechanism of ILK remain unclear in tumor.

Ribonuclease A (RNase A), a member of superfamily, has strong catalytic capability to the hydrolysis of RNA. When the protein reaches an optimal level, it might act as a passage of genetic information from DNA to protein via RNA. However, the protein at a higher concentration causes cell damage by resisting protein synthesis through increased ribonucleolytic activity. And this phenomenon aroused great interest of scientists in designing ribonuclease inhibitors [12–14]. Ribonuclease inhibitor (RI) is a 50 kDa horseshoe-shaped protein. It functions as the inhibitor of the ribonucleolytic activity of RNase A and angiogenin via a close interaction with them [15–18]. RI, also termed angiogenin inhibor or RNH1, is abundant in the cell cytosol and also exits in mitochondria and nuclei [19]. The capacity of RI to protect genome integrity and cell viability from oxidized stress damage has been documented, and corresponds with the sensitivity to oxidation and the role of the protein in reactive oxygen species (ROS) scavenger in vitro [20–22]. RI, made up of 15 leucine-rich repeats (LRRs), emerges as a core protein in the research of LRRs. Its unique structure and function suggested that it could have a significant role in protein-protein interaction [23]. Therefore, we suppose that unknown biological functions exist in RI.

Human ILK gene and RNH1 gene both located at chromosome 11p15. In chromosome 3 of Drosophila, chromosome 7 of mice, chromosome 1 of rat and chromosome 11 of human, RI and ILK are always neighboring in the same chromosome. Depending on the chromosome proximity method, if the neighborship of two genes is conserved in different genomes during revolution, they may have some related functions [24]. We have reported that ILK and RI had a co-localization and are negatively correlated in the EJ cells [25]. Our previous experiments also showed that RI down-regulated ILK and suppressed the phosphorylation of PKB/Akt and GSK-3β [26]. Therefore, these data suggest the possibility of the interaction between the two proteins. However, it’s unclear whether RI can directly bind to ILK. Furthermore, mechanism underlying the interaction between the two proteins has not been reported so far.

This research is aimed at further revealing the molecular mechanisms of interplay between ILK and RI as well as supplying new methods to the therapy of carcinoma of bladder. Here, we report that ILK could combine with RI to regulate its biological function in vivo and in vitro. This research first proved that direct binding of RI and ILK regulated EMT through ILK signaling pathway in bladder cancer. Our study may provide novel prognostic and therapeutic targets for bladder cancer.

## Methods

### Cell lines, animal and reagents

EJ cells, HEK 293 cells, pcDNA3.1(−)-myc-RI, pGEX-4 T-RI and pEGFP-C1-RI plasmids were conserved and prepared by our laboratory. pGEX-4 T-1 was bought from GE Healthcare China. pEYFP-N1vector was from Clontech. pCMV-3xflag-CMVTM-10 was purchased from Sigma. BALB/C nude (nu/nu) mice were obtained from Beijing HFK Bioscience Company (Beijing, PR China). FBS was from TBD Science (Tianjin, PR, China). DMEM/High glucose medium, RPMI 1640 medium and G418 were purchased from Gibco-BRL (Carlsbad, CA, USA). Lipofectamine 2000 was bought from Invitrogen, Inc., (Carlsbad, California). Monoclonal mouse antibody of anti-human ILK was purchased from Santa Cruz Biotechnology (Santa Cruz, CA, USA). Rabbit anti-human β-actin, CD31 antibody, Monoclonal primary rabbit antibody of anti-human PI3K, p-PI3K, PTEN, p- PTEN, Akt, p-Akt, GSK3β, p-GSK3β, mTOR, p-mTOR and β-catenin were obtained from Bioworld Technology, Inc. (St. Louis, USA). The rest of the primary antibodies are from Proteintech Group, Inc (Chicago, IL, USA). Cell Counting Kit-8 was bought from Genview Scientific, Inc (Craigieburn, VIC, AUS).

### Construction of the plasmids

Human ILK cDNA sequence (accession number: NM_004517) was provided by the GenBank. The pEYFP-ILK and pCMV-3 × Flag-ILK plasmids were constructed through the application of recombinant-DNA technology. The design for pEYFP-N1 plasmid (Clontech) primers was as follows: forward, 5′-CCG*CTCGAG*GCTATGGACGACATTTTCAC-3′, and reverse, 5′- CG*GAATTC*TCTTGTCCTGCATCTTCTCA -3′; The primers for pCMV-3xflag-CMV™-10 plasmid (Sigma) is following: forward, 5′- CCC*AAGCTT*ATGGACGACATTTTCACTC -3′, and reverse, 5′- GC*TCTAGA*CTACTTGTCCTGCATCTTC -3′. The recombinant plasmids pEYFP-N1-ILK was identified by using restriction enzymes Xho I and EcoR I (underlined), and the pCMV-3 × Flag-ILK was identified by using restriction enzymes Hind III and Xba I (Italic). Finally, all plasmids were sequenced and then using BLAST software to verify the sequences.

### Cell culture and establishment of stable cell lines

The EJ cells were cultured in RPMI 1640 media with 10 % FBS in a culture chamber containing 5 % CO_2_ at 37 °C. The cells were collected and then inoculated into six well plates. Calcultate the growth rate of the cells and enable them to grow until 80–90 % of the confluence on the next day. The cells were immediately transfected with the plasmids by Lipofectamine 2000. After 2 days, the choice was executed with 800 μg/ml of G418 for 2 weeks and 400 μg/ml of G418 for another 2 weeks. Finally, we selected the positive clones, and then proliferated stable cell lines that express ILK and the blank control vector which named EJ-ILK and EJ-FLAG cell respectively. Recombinant lentivirus containing RI (LV-RI) and control (LV-NC) were purchased from Western Biotechnology (Chongqing, PR, China). In order to establish the stable cell line, 2 × 10^5^ EJ cells were transfected with 4 × 10^6^ transducing units of lentiviruses, the supernatant was removed after 24 h and replaced with complete culture medium. After that, the EJ cells were selected with 2 μg/ml puromycin for two weeks. The two kinds of stable cell lines were named EJ-RI and EJ-LV5, respectively. HEK 293 cells were cultivated in DMEM/High glucose medium with 10 % FBS at 37 °C.

### Western blot analysis

Immunoblot analysis was performed as following. Briefly, total cell protein lysates were obtained with RIPA buffer containing proteinase inhibitor (Sigma, St. Louis, MO, USA). The proteinof 30 μg was separated by appropriate concentration of SDS PAGE and shifted to PVDF membranes, then blocked, the membranes were incubated overnight with primary antibodies of AKT, p-AKT, GSK3β, p-GSK3β, PI3K, p-PI3K, PTEN, p-PTEN, mTOR, p-mTOR, β-catenin, E-cadherin, MMP2, MMP9, N-cadherin, Vimentin, Twist, Snail, S100A4, Smad2 and β-actinat at 4 °C. Next, washed with TBST, incubated with species-specific secondary antibodies labeled with HRP at 37 °C for 2 h and visualized with an ECL chemiluminescent detection system (Thermo Scientific). Loading differences were normalized using a monoclonal β-actin antibody.

### GST pull down

The encoded RI-GST fusion proteins and the control GST proteins were expressed in BL21 cells after induction with IPTG. The GST-fusion proteins were arrested by glutathione–Sepharose 4B beads (GE Healthcare, Little Chalfont, UK) for the GST pulldown assays, and Flag-ILK (transfected EJ cells or HEK293 cells extracts) was added. Binding was performed for 3 h under rotation at 4 °C and the beads were washed with ice-cold PBS. The supernatant was loaded to gels followed by Western blot identification.

### Co-immunoprecipitation assay (Co-IP)

For co-immunoprecipitation, EJ cells and HEK 293 cells were co-transfected with pCMV-3 × Flag-ILK and pcDNA3.1(−)-myc-RI using Lipofectamine 2000 reagent (Invitrogen). Cells were trypsinized and rinsed in PBS. The protein extracts were then incubated using anti-Myc antibody and precipitated with protein A-agarose (Invitrogen). The precipitated proteins were separated and detected with Western blot using anti-RI or anti-ILK antibodies. Finally, the blots were visualized using ECL system (Amersham Biosciences).

### Confocal microscope scanning and fluorescent resonance energy transfer (FRET) assay

To visualize interaction of ILK and RI in EJ cells, FRET imaging was conducted using cy3-conjugated goat anti-rabbit IgG and Alexa Fluor 488- conjugated goat anti-mouse IgG as biosensors. EJ cells were transfected with pCMV-3 × Flag-ILK and pcDNA3.1(−)-myc-RI. And then conventional immunofluorescent assay was performed. Images of 488-ILK and cy3-RI FRET activities were acquired using a Leica confocal imaging spectrophotometer system (TCS-SP, Germany). Briefly, the fluorescence images for 488-ILK and cy3-RI were collected repetitively at a 10 s interval for five times. After that, cy3 was photobleached with full power of 543 nm line laser. The emission intensities of 488-ILK and cy3-RI were monitered and recorded. The FRET efficiency (FRETe) was determined by computing the percentage of the 488-ILK fluorescence recovery [FRETe = (488-ILKA −488-ILKB)/488-ILKA × 100 %], where 488-ILKA was the 488 intensity after photobleaching and 488-ILKB was the 488 intensity before photobleaching of cy3.

### Co-colonization detection and immunocytofluorescence

EJ cells and 293 cells were co-transfected with plamids pEYFP-N1-ILK and pEGFP-C1-RI using lipofection. The fluorescence proteins were visualized with Olympus multifunction microscope (Tokyo, Japan) equipped with appropriate filters (Chroma). Finally, images were captured and processed. The other way to detect location: EJ cells were co-transfected with plasmids pCMV-3 × Flag-ILK and pcDNA3.1(−)-myc-RI. After that, cells were fixed by 4 % paraformaldehyde, then permeabilized by Triton X-100. For detection of co-location, an antibody mix containing ILK (Santa Cruz, CA, USA) and RI was used. In co-immunostainings, goat anti-rabbit conjugated Alexa 594 and goat anti-mouse conjugated Alexa 488 were used and nuclei was stained with DAPI. Observations were performed and pictures were taken under Laser Scanning Confocal Microscope. For conventional IF assays, cells were incubated overnight at 4 °C in primary antibodies against ILK (Santa Cruz, CA, USA) and RI separately. And the IF was performed as described above.

### Cell morphology, cell cycle and cell growth

The cells were grown on cover glass in 6-well plates, washed with PBS. The pictures of cells were taken under phase contrast inverted microscope (NIKON TE2000-U, Japan). For cell cycle assay, cells were trypsinized, washed by PBS, and then resuspended with 70 % ethanol and incubated on ice for 24 h. The distribution of cell cycle phase was assayed by flowcytometry (Becton Dickinson FACSCalibur, USA). Cell proliferation rate was determined by CCK-8 assay. Briefly, cells were seeded in 96-well plates. The cell proliferation was monitored every 24 h by CCK-8 assay. The medium were replaced with fresh medium containing 10 % CCK-8, shake the plates gently and then incubate them in 37 °C for 3.5 h. The absorbance value was detected by a plate reader at 450 nm. ALL of the assays above were repeated 3 times.

### Mouse xenograft models, histology, immunohistochemistry and immunofluorescence

BALB/C nude mice used in these investigations were handled in accordance with the Guide for the Care and Use of Laboratory Animals (National Research Council, 1996). The animal production license and animal license of Chongqing Medical University are SCXK (Beijing) 2014–0004, SCXK (Chongqing) 2007–0001. The stable–transfected EJ cell lines (5 × 10^6^) mixed with 1 × PBS were injected into the flank of nude mice (4-week-old) subcutaneously. Tumors and lungs were harvested 30 days after injection, the tumor tissue was weighed. The 4 μm tumor tissue sections were stained by HE (hematoxylin-eosin) and microvessels were numbered. The 5-μm-thick adhesion slides were dewaxed and then immersed in a gradient alcohol and washed three times. They were treated with 0.3 % H_2_O_2_ and then subjected to 10 % sheep serum for 30 min. The slides were incubated overnight with primary antibodies of RI, ILK, p-AKT, p-GSK3β, p-mTOR, β-catenin, E-cadherin, MMP2, MMP9 and Vimentin. The next day, slides were incubated with secondary antibodies of Anti-mouse and rabbit IgG for 1 h and then immersed in avidin biotin-peroxidase complex diluted in PBS and visualized with amino-ethyl carbazole chromogen stock solution. For IF, 10-μm-thick frozen tumor sections were fixed in acetone, blocked, and incubated overnight at 4 °C in primary antibodies against p-AKT, p-GSK3β, p-PI3K, p-PTEN, p-mTOR, β-catenin, E-cadherin, MMP2, MMP9, Vimentin, S100A4 and CD31. The next day, sections were incubated with fluorescein 594 and 488-conjugated secondary antibodies. Images were obtained using Olympus multifunction microscope (Tokyo, Japan).

### Analysis of ILK and RI expression in tumor tissue from nude mice and human bladder cancer samples

Bladder carcinoma and corresponding non-tumor normal tissues were obtained from the first clinical college of Chongqing Medical University. All samples were de-identified. The 10-μm-thick frozen tumor tissue sections were fixed with acetone, blocked, and incubated overnight at 4 °C in primary antibodies against ILK and RI. As described above, HE staining and IF assay were conducted.

### Statistical analysis

Data were processed with SPSS19.0 statistical software. The values were expressed as means ± SD. Student’s t tests were used for statistical analysis. *P* values of less than 0.05 were considered to be statistically significant.

## Results

### Over-expression of ILK and RI is identified

**Fig. 1:**
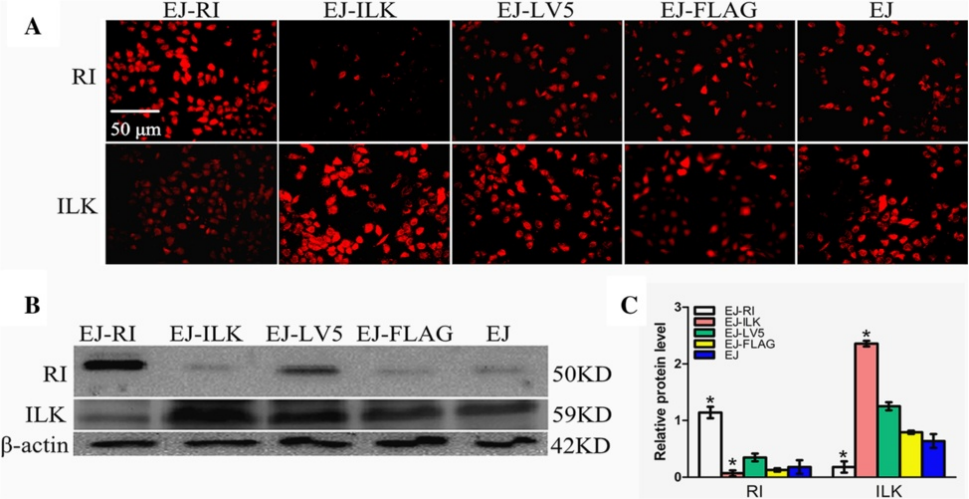
ILK and RI expression is determined by Western blot and Immunofluorescence after transfection for 48 h. **a** Immunofluorescent observation of ILK and RI was detected respectively. EJ-ILK cells demonstrated remarkably stronger immunofluorescent signal in cytoplasm whereas EJ-RI cells demonstrated significantly stronger immunofluorescent signal in nucleus, compared with the other control groups respectively (magnification × 200). **b** & **c** ILK and RI protein expression level was evaluated by Western blot. Proteins were separated by SDS-PAGE and then binded to specified monoclonal ILK antibodies or polyclonal RI antibodies. β-actin was used as control. The levels of ILK proteins were remarkably increased by transfected with pCMV-3 × FLAG-ILK and the levels of RI proteins were significantly increased by transfected with lentivirus containing RNH1 (LV-RNH1), compared with the control groups respectively

ILK gene sequence and vector were verified correctly by enzyme digestion, sequence analysis (data not shown). The transfected cells were selected, and then cloned, proliferated, finally verified by Western Blot and immuno-fluorescence assay. The expression of RI protein levels was significantly heightened in EJ-RI cells, compared with the other two control group cells respectively. The expression of ILK was observably increased in EJ-ILK cells, compared with the control group cells respectively (Fig. 1a). Immunofluorescence assay revealed that RI and ILK were brighter in EJ-RI and EJ-ILK cells respectively, compared with the corresponding control cells (Fig. 1b and c). The results demonstrated that RI or ILK were steadily expressed in the cells respectively.

### ILK binds to RI in vivo and in vitro

**Fig. 2:**
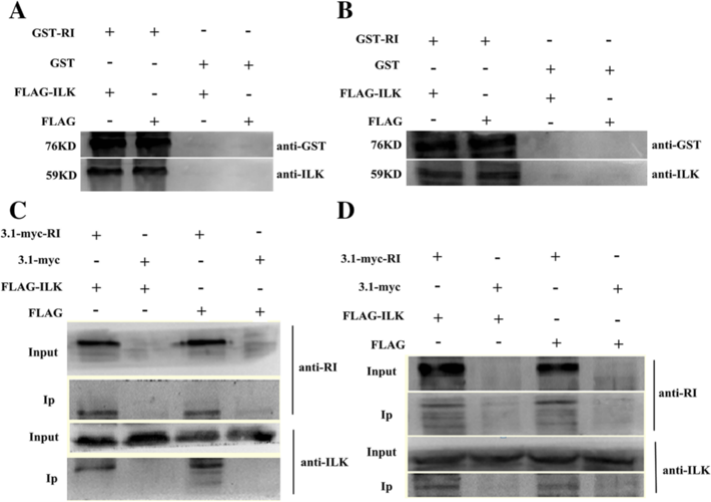
RI interacts with ILK in vivo and in vitro. The interaction of RI with ILK was detected as described in “Materials and methods” with GST pulldown and co-immunoprecipitation (Co-IP). **a** & **b** The interaction of RI with ILK was determined by GST pull down. The bacteria were induced with 0.1 mmol/L IPTG in different time, and then the fusion protein was obtained after ultrasonic decomposition and washed repeatedly. Western blot showed that ILK protein from EJ cells (**a**) or 293 cells (**b**) transfected with pCMV-3 × flag-ILK and pCMV-3 × flag could be combined with GST-RI and be specifically pulled down. **c** & **d** RI was co-immunoprecipitated with ILK in EJ cells (**c**) or 293 cells (**d**). The interaction of RI with ILK was detected as described in Methods, the co-immunoprecipitation of RI and ILK suggested that ILK could specifically bind to RI in the cells

To determine whether there is a direct interaction between ILK and RI, in vitro pull-down experiments were conducted. GST-RI constructs were used in pull-down assays with plasmids pCMV-3 × flag-ILK. Western blot proved that ILK protein from EJ cells (Fig. 2a) and 293 cells (Fig. 2b) transfected pCMV-3 × flag-ILK and endogenous ILK could be captured by GST-RI and be pulled down specifically, demonstrating a physical binding of RI and ILK in vitro.

To further investigate the interplay of RI and ILK, we executed co-immunoprecipitation detection. RI and ILK were explored in immunoprecipitation complex with anti-myc antibodies. The results demonstrated that ILK and RI could have a binding and interaction (Fig. 2c and d).

### Fluorescent resonance energy transfer and colocalization of ILK with RI are identified

**Fig. 3:**
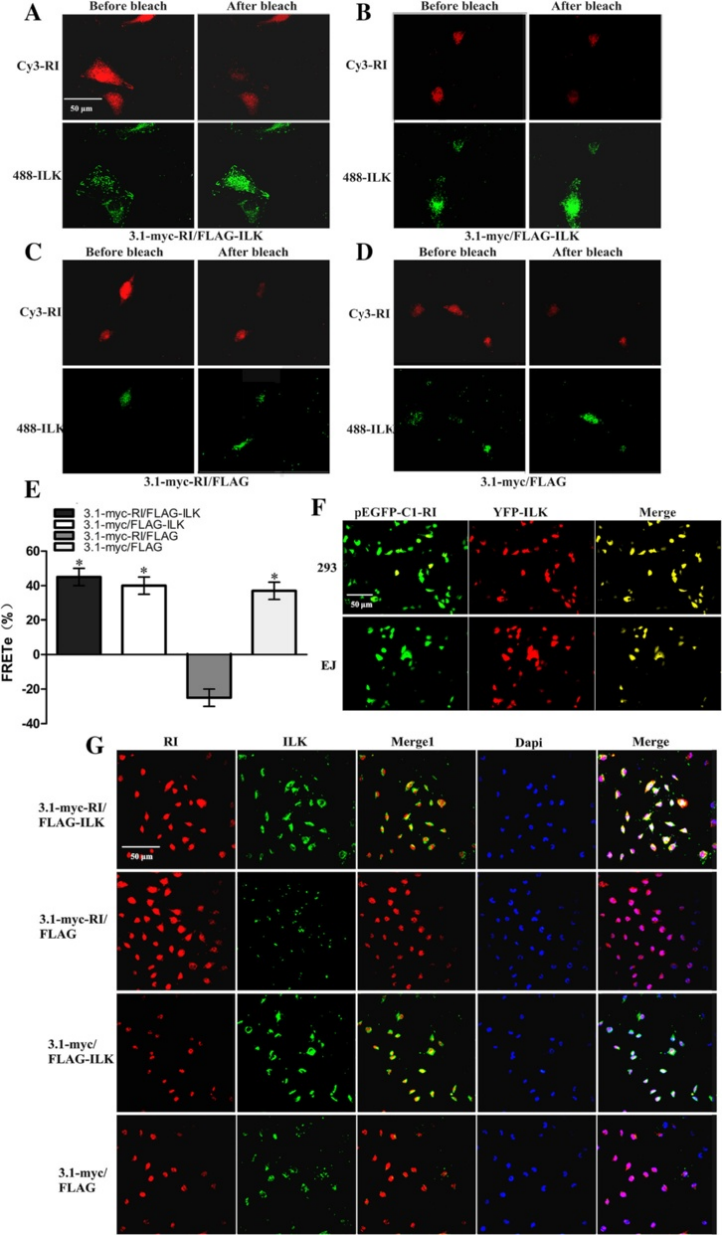
Fluorescent resonance energy transfer and colocalization of ILK with RI are assayed. **a**-**d** EJ cells were co-transfected with plasmids as described previously, and then followed by photobleaching analysis. Images indicated the 488 and cy3 fluorescence emission intensities in the cells before and after photobleaching. **e** The FRET efficiency (FRET_E_) was calculated as the percentage of the 488 fluorescence recovery. The experiments were repeated 3 times. **f** The Intracellular localizations observation of RI and ILK. EJ cells and 293 cells were co-transfected with plamids pEYFP-N1-ILK and pEGFP-C1-RI. The two Proteins labeled with fluorescent proteins revealed a colocalization under Olympus multifunction microscope. **g** RI expression and colocalization experiment also were observed under laser scanning confocal microscope. Immunofluorescence staining of RI (*red*) and ILK (*green*) in four groups of EJ cells co-transfected with different pairs of plasmids. The nucleus was stained with DAPI (*blue*). The yellow part demonstrated that ILK colocalized with RI in EJ cells. The expression of RI markedly decreased in ILK-upregulated cells compared with the control group cells (magnification × 200)

To further detect real-time dynamic ILK-RI interaction in the living cell physiological conditions, we then applied FRET technology. As shown in the Fig. 3a, b, c and e, the FRET appeared between ILK and RI in EJ cells, which further confirmed the interaction between ILK and RI in living cells.

To investigate whether RI and ILK would localize in the same site of live cells for functional partners, we used vectors of GFP-RI fusion proteins and YFP-ILK fusion proteins. The subcellular localization of exogenous ILK and RI was observed in 293 and EJ cells under immunofluorescence microscopy. RI was green while ILK was red in both 293 cells and EJ cells. Then, we acquired the yellow merge figures (Fig. 3f). Furthermore, we performed IF assay to detect the localization. The immunofluorescence revealed that most of ILK distributed in cytoplasm (green). We also observed that RI was mainly spread in the cytoplasm (red). As a result, the yellow area which demonstrated that co-localization of ILK with RI existed in the cytoplasm (Fig. 3g).

### Up-regulating RI or ILK affects cell morphology, cell cycle and proliferation

**Fig. 4:**
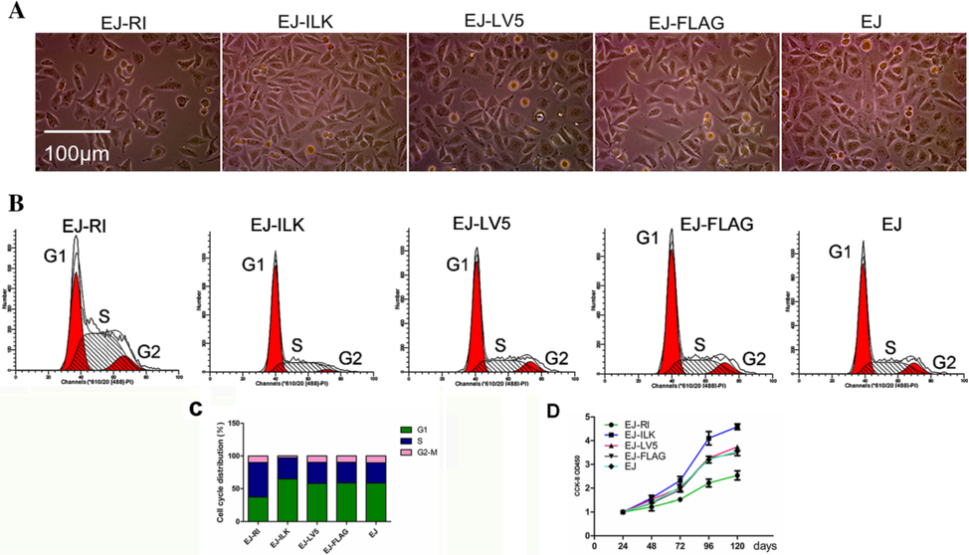
Cell morphology, cell cycle and cell proliferation are observed. **a** The observation of living cell was executed (magnification × 200). The EJ-ILK group’s cells showed mesenchymal a characteristic and more division phases compared with some cells in the other control groups. On the contrary, EJ-RI cells displayed an epithelial morphology and less division phases compared with some cells in the other control groups. **b** Flow cytometry was conducted with propidium iodide staining. Experiments were repeated three times, samples were analyzed by Student’s *T*-test and statistical significance with *P < 0.05. **c** Flow cytometry analysis of cell cycle distribution demonstrated that the percentages of the EJ-RI cells in S phase were significantly increased, whereas the percentages of EJ-RI cells in G1 phase were decreased in comparison to EJ cells or EJ-LV5 cells. In addition, a slight increase in G1 phase and a decrease in G2/M phase were detected in EJ-ILK cells. **d** 1.5 × 10^3^ cells were added into a 96 well plate, the CCK8 assay was performed and recorded the A450 with a plate reader every 24 h. Each point represents the mean of twelve independent samples and experiments were repeated 3 times. EJ-RI cells showed inhibited proliferation abilities, whereas increased proliferation abilities were detected in EJ-ILK cells, compared with control group cells respectively, *P < 0.05

Live cells images showed that EJ-ILK cells became mesenchymal morphology and higher malignant phenotypes such as a larger nucleus and more division phases compared with other two control groups. Whereas, the cells in EJ-RI group revealed epithelial features and lower degree of malignant morphology (Fig. 4a).

The cell cycle was detected by flow cytometry. The graph displayed that the percentage of EJ-RI cells was increased by 71.24 % ± 6.89 % and 63.26 % ± 7.71 % in the S phase as well as it was decreased by 36.18 % ± 4.26 % and 35.82 % ± 3.66 % in the G_1_ phase, compared with the EJ cells and EJ-LV5 cell group, respectively. Meanwhile, the percentage of EJ-ILK cells was increased by 10.73 % ± 1.10 % and 10.41 % ± 1.70 % in the G_1_ as well as it was decreased by 72.58 % ± 6.23 % and 70.08 % ± 10.40 % in the G_2_/M phase, compared with the EJ cells and EJ-FLAG cell group, respectively. However the EJ-RI in G_2_/M phase and EJ-ILK in S phase did not show significantly different. The results above showed that the growth of EJ-RI cells was prohibited with G_1_ phase decreasing and S phase arresting while ILK induced G_2_/M phase reducing and G_1_ phase increasing in the EJ cells (Fig. 4b and c).

The 450 nm absorbance is in accordance with the number of living cells. The Inhibition rates of EJ-RI cells to EJ vector cells, and the promoting rates of EJ-ILK to EJ vector cells were 29.41 and 35.29 % at 120 h respectively. Whereas, the growth of the control cells was not be affected, *P < 0.05. The A450 was observably decreased with CCK8 in the EJ-RI cells. The EJ-RI cells demonstrated lower cell proliferations than those of the EJ cells and the EJ-LV5 cells. As for EJ-ILK cells, the A450 was significantly increased with CCK8, compared with EJ cells and EJ-FLAG cells (Fig. 4d). According to the data, the reproductive capacity of the EJ-ILK cells strengthened obviously.

### Up-regulating RI or ILK affects the expressions of proteins associated with ILK signaling pathways and EMT

**Fig. 5:**
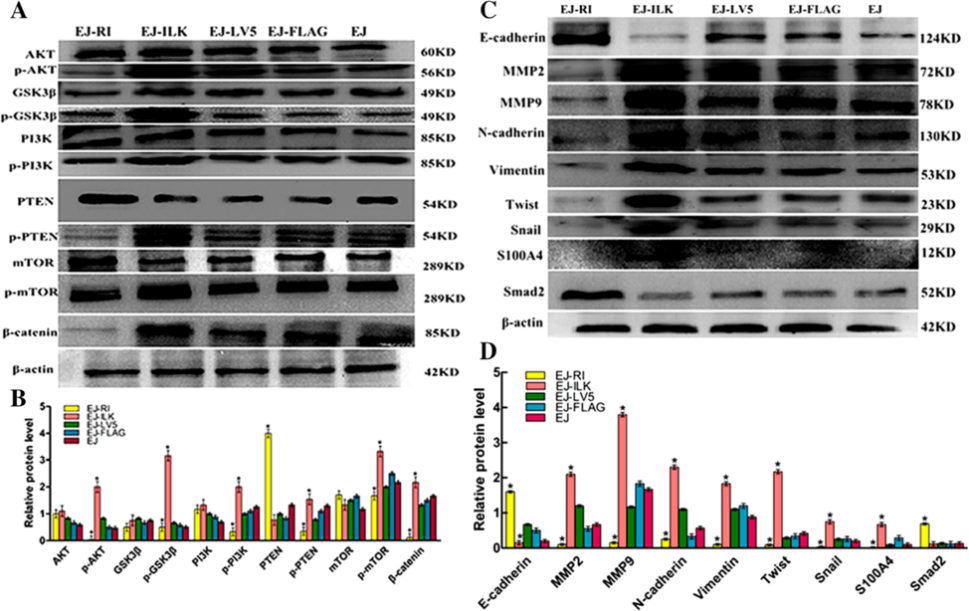
Up-regulating RI and ILK regulates the expression of ILK signaling pathway molecules and proteins associated with EMT in vitro. The total protein of cells was collected using cell lysis buffer, the expressions of ILK signaling pathway molecules and proteins associated with EMT were examined using corresponding antibodies by immunoblot analysis. β-actin was served as the control. Relative protein levels were normalized against those of β-actin by MJ Opticon Monitor Analysis Software (Bio-Rad). Data were calculated as means ± S.D (*n* = 3). *P < 0.05. **a** & **b** The results demonstrated that transfections with pCMV-3 × FLAG-ILK in EJ cells significantly up-regulated the expressions of p-Akt (S473), p-GSK3β (S9), p-PI3K, p-PTEN, p-mTOR and β-catenin, compared with control groups respectively, whereas these protein levels decreased obviously in EJ-RI cells. The protein expressions of AKT, PI3K, mTOR and GSK3β did not change apparently, compared with control groups respectively. **c **& **d** Western blot assay and semi-quantitative analysis showed that protein levels of MMP-2, MMP-9, N-cadherin, Snail, Twist and Vimentin were decreased, while protein levels of E-cadherin and Smad2 were significantly increased in EJ-RI cells compared with the control groups respectively. E-cadherin was significantly weaker in the EJ-ILK cells group compared with the other control groups and MMP-2, MMP-9, N-cadherin, Snail, Twist, S100A4 and Vimentin were increased in EJ-ILK cells groups

To futher investigate the molecular mechanism of RI and ILK in cell metastasis, we detected protein levels of theimportant target molecules of ILK pathway and EMT with Western blot. The expressions of AKT, GSK3β, PI3K and mTOR and did not significantly change. But, the levels of p-Akt, p-GSK3β, p-PI3K, p-PTEN, p-mTOR and β-catenin were increased markedly in EJ-ILK cells whereas these protein levels decreased obviously in EJ-RI cells (*P < 0.05). MMP2, MMP9, N-cadherin, Vimentin, Twist, Snail and S100A4 were increased significantly in EJ-ILK cells whereas these protein levels decreased obviously in EJ-RI cells, compared with control group cells respectively (*P < 0.05). Furthermore, PTEN, E-cadherin and Smad2 expressions were obviously increased in the EJ-RI cells group, whereas these protein levels were down-regulated in the EJ-ILK cells group, compared with control groups respectively (Fig. 5a, b, c and d). Results show that the RI increase might be related with inhibition of the ILK signaling pathways and EMT. While, up-regulating ILK promoted ILK signaling pathways and EMT.

### Up-regulating RI or ILK affects tumor growth and experimental lung metastasis of BALB/C nude mice

**Fig. 6:**
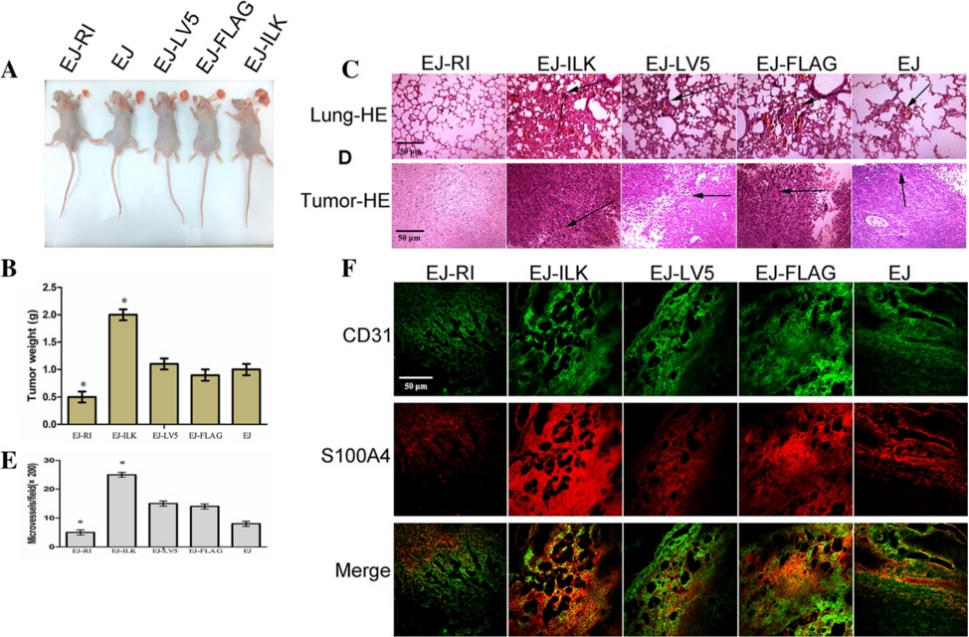
Up-regulating RI and ILK modulates tumor growth and spontaneous lung metastasis of BALB/C nude mouse xenograft model. 5 × 10^6^tumor cells including the EJ-RI cells, the EJ-ILK cells, EJ-LV5 cells, EJ-FLAG and EJ cells were respectively injected subcutaneously into the flanks of the BALB/C nude mice. After 4 weeks, the mice were ultimatedly killed. The tumors and lungs were collected, weighed, and photographed. **a** Representative images of the xenograft tumors and the BALB/C nude mice after removing tumors. **b** Tumor weight analysis. The EJ-ILK cell groups remarkably promoted the growth of xenograft tumor compared with the control groups, whereas EJ-RI cells groups inhibited the growth of xenograft tumor, compared with the other control groups. **c** HE staining of lung sections, arrows show invasive tumor cells in lung. (magnification 200×). **d** HE staining of tumor sections, arrows indicates microvessels among xenograft tumors (magnification 200×). **e** Microvessel density analysis. **f** Immunofluorescent staining with antibodies against CD31 and S100A4 of vascular endothelial cells. Immunofluorescent and histochemical study demonstrated that large amounts of microvessels could be detect in the tumors of the mice injected with EJ-ILK cells. By comparison, there were fewer microvessels in the tumor of the mice injected with EJ-RI vector cells (magnification × 200)

To further study to the effects of the RI and ILK on tumor growth respectively, 5 × 10^5^ various kinds EJ cells were inoculated subcutaneously into nude mice. HE staining and IF assay were conducted to further verify the influence of RI and ILK on tumor growth and metastasis. The rates of tumor formation were significantly lower in EJ-RI group compared with the other two control group cells, respectively. The rates of inhibitory tumors were 54.54 and 60.11 % compared with the EJ-LV5 and EJ cells groups. The tumors in the EJ-ILK cells groups were heavier than those in the other two control groups. The promoting rates of tumors were 222.22 and 200.94 % compared with the EJ-FLAG and EJ cells groups, respectively, *P < 0.05 (Fig. 6a and b). The results showed that the ILK significantly facilitated the growth of bladder cancer, whereas, RI obviously suppressed xenograft tumor. The mice injected with EJ-RI cells displayed an obvious suppression of the metastasis with a substantial reduction of invasive tumor cells. Mice injected with EJ-ILK cell groups showed a remarkable increase of pulmonary metastatic nodules, compared with EJ and EJ-FLAG cells group (Fig. 6c). Microvessels of 5 different fields were counted under microscope with the HE sections of tumor. EJ-ILK cells reaveled more tumor microvessels compared with the control groups, whereas EJ-RI groups indicated fewer microvessels compared with the controls (*P < 0.05) (Fig. 6d and e). Immunofluorescent analyses of CD31 and S100A4 were conducted. Results displayed that the EJ-ILK cells group had a higher CD31 and S100A4 expression and apparent increase of angiogenesis; whereas weaker CD31 and S100A4 expression and fewer microvessels were observed in the EJ-RI groups (Fig. 6f).

### Up-regulating RI or ILK affect the expressions of proteins associated with ILK signaling target molecules and EMT in tumor tissue

**Fig. 7:**
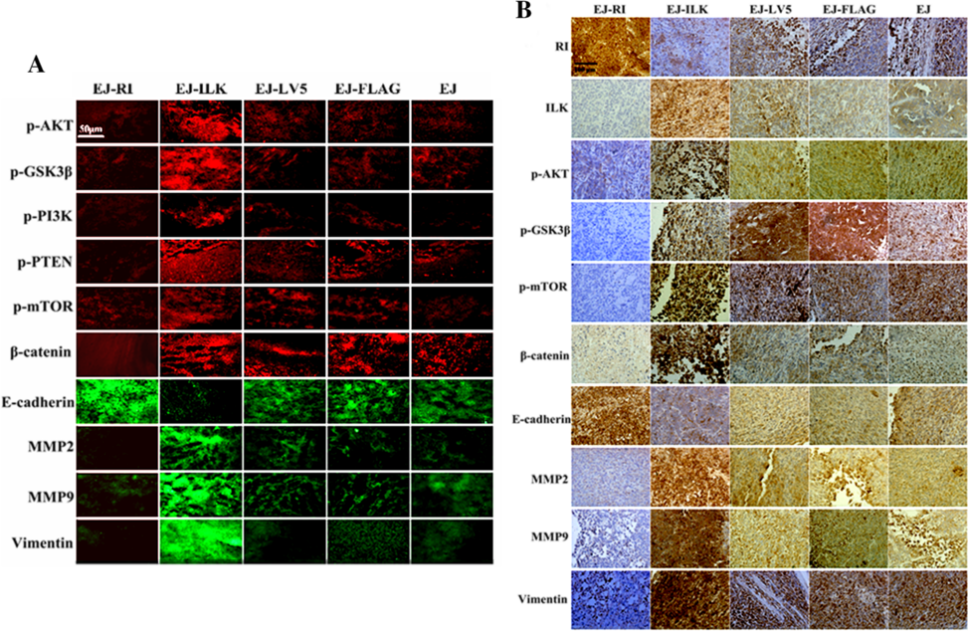
Up-regulating RI and ILK regulates the expressions of ILK signaling pathway molecules and EMT-related proteins in vivo. **a** Immunofluorescent sections were stained in tumor xenograft tissue with rabbit anti-human p-Akt (S473), p-GSK3β (S9) p-PI3K, p-PTEN, p-mTOR, β-catenin, E-cadherin, MMP2, MMP9 and Vimentin respectively, then were incubated with Alexa Fluor 594 Goat Anti-Rabbit IgG or Alexa Fluor 488 Goat Anti-Rabbit IgG secondary antibody. EJ-ILK cell groups resulted in significantly higher p-Akt, p-GSK3β, p-PI3K, p-PTEN, p-mTOR, β-catenin, MMP2, MMP9 and Vimentin as well as lower E-cadherin expressions in tumor tissue, compared with EJ and EJ-FLAG cells groups. In contrast, weaker p-Akt, p-GSK3β, p-PI3K, p-PTEN, p-mTOR, β-catenin, MMP2, MMP9 and Vimentin expression were detected in tumor tissue of the EJ-RI cell groups along with stronger E-cadherin expressions, which was in accordance with experiments in vitro (magnification × 200). **b** Immunohistochemical sections stained with mouse anti-human ILK, rabbit anti-human RI, p-Akt(S473), p-GSK3β(S9), p-mTOR, β-catenin, E-cadherin, MMP2, MMP9 and Vimentin respectively, the nuclei were counterstained by hematoxylin (magnification 400×). Representative images demonstrated that the EJ-RI cell groups had a weaker brown immunostain for ILK, p-Akt, p-GSK3β, p-mTOR, β-catenin, MMP2, MMP9 and Vimentin as well as a stronger positive signal for RI and E-cadherin in cytoplasm compared with the control group cells respectively. EJ-ILK cells group revealed much lower RI and E-cadherin expressions as well as stronger positive signal of ILK, p-Akt, p-GSK3β, p-mTOR, β-catenin, MMP2, MMP9 and Vimentin expressions in tumor tissue (magnification × 400)

Immunohistochemical and immunofluorescent detections were conducted to determine the impacts of ILK and RI expression on ILK signaling pathways and EMT in tumors respectively. The results revealed that EJ-ILK cells group showed much lower RI and E-cadherin levels as well as stronger positive signal of ILK, p-Akt, p-GSK3β, p-PI3K, p-PTEN, p-mTOR, β-catenin, MMP2, MMP9 and Vimentin in tumor tissue, whereas lower ILK, p-Akt, p-GSK3β, p-PI3K, p-PTEN, p-mTOR, β-catenin, MMP2, MMP9 and Vimentin expression were observed in tumor tissue of the EJ-RI groups (Fig. 7). These results were consistent with analysis in vitro*,* suggesting that the interaction between RI and ILK could be related with regulation of ILK signaling pathway and EMT.

### High ILK and low RI protein levels are observed in tumor tissue of nude mice and human bladder cancer

**Fig. 8:**
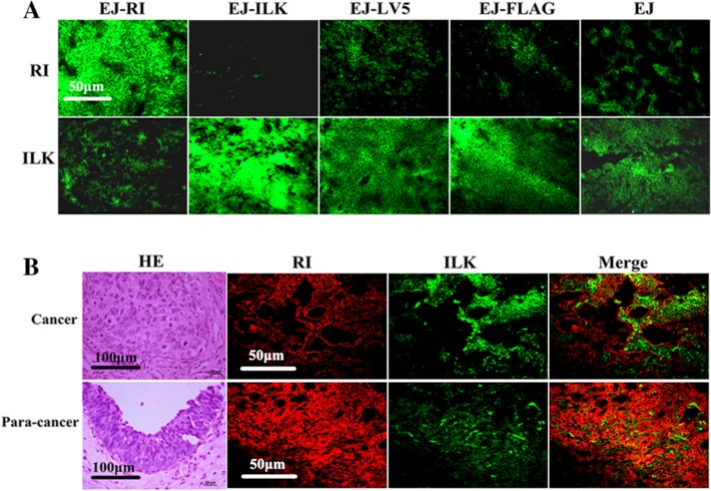
Expressions of ILK and RI in tumor tissue from nude mice and human bladder cancer are detected. **a** Tumor tissues from nude mice were stained with mouse anti-human ILK and rabbit anti-human RI antibodies separately. **b** HE staining of human bladder tissue sections were conducted and photographs were taken under microscope (magnification × 400). Human bladder tumor tissue and adjacent normal bladder tissue were incubated in the antibodies mixed with mouse anti-human ILK and rabbit anti-human RI antibodies. Bladder tumor tissue from both nude mice and human showed higher ILK expression as well as lower RI expression, whereas the expressions of RI and ILK in adjacent normal bladder tissue were just the opposite (magnification × 200)

To gain insights into the expression of ILK and RI in tumor tissue from nude mice and human bladder cancer, we performed immunofluorescent assay to determine the two kinds of protein level in the specimens. The data suggest that ILK expression was negatively correlated with RI levels in the bladder cancer specimens from nude mice and human. Human tumor and nude mice xenograft tumor tissues showed the expression of ILK was increased while the expression of RI was inhibited (Fig. 8a and b). The fact that higher level of RI and lower level of ILK in paired normal tissues suggest that RI could be required to regulate ILK for suppression of bladder cancer.

## Discussion

Invasive or metastatic bladder cancer is a lethal disease in spite of surgery, radiation treatment and chemotherapy. Unfortunately, transurethral resection or other adjuvant treatments do not achieve much in preventing tumor metastasis and recurrence. Finally, 20–30 % of those tumors progress into more aggressive tumors and cause death of patients [27, 28].

Protein-protein interaction plays a critical role in every essential biological process. Human ribonuclease inhibitor (RI) contains 15 Leucine-rich repeats (LRRs). Such repeats have been considered to have multiple functions, such as extracellular matrix interaction, cell-cycle regulation, DNA repair and enzyme inhibition [17]. Therefore, we speculate that RI might still have some undiscovered biological roles. Our previous study showed that ILK and RI have a common subcellular location, and the expression of RI was negatively correlated with ILK in BIU-87 cells [29]. Yet, correlation between RI and ILK has not been fully understood. ILK plays an important role in transducting many of the biochemical signals that are triggered by cell-matrix interactions and modulate fundamental processes such as growth, survival, differentiation, invasion and angiogenesis. In vertebrates, loss-of-function analyses have highlighted the importance of ILK in the regulation of protein-protein interactions that are related to the activation of signaling pathways. The further study of ILK will emphasize on its function of the direct and indirect interactions existing in ILK. Which interaction is more important for normal tissue depends on which interaction is more crucial for the development of human malignant tumors. As a matter of fact, if ILK could be effectively targeted, the upper proposition will be proved and valued properly [30–32]. However, it is unknown whether there is also an interaction between ILK and RI in cancer progression and the mutual relationship has remained unclear so far.

Here, we first reported that RI and ILK have a physical interaction between them, and we use colocalization analysis, co-immunoprecipitation and pull-down assays to prove the hypothesis. We also found for the first time that RI could suppress ILK activity by extremely tight combination in vitro. RI contains leucine-rich repeats (LRRs), which have vast surface areas to supply protein interaction [23].

In this study, we also showed that ILK could promote the cell proliferation, change cell morphology and regulate cell cycle. Meanwhile, we demonstrated that over-expression RI decreased the proliferation and led to cell cycle arrest in S phase. Moreover, we also showed that over-expression of ILK remarkably promotes the phosphorylation of signaling targets AKT, GSK-3β, PI3K and mTOR in vivo and vitro. ILK/AKT/mTOR signaling pathway is widely known for modulating cell growth, differentiation, apoptosis, cellular metabolism and cancer cell survival. ILK phosphorylated Akt to play an important role in anti apoptosis through direct phosphorylation of downstream target protein; ILK also phosphorylate GSK-3 to inhibit its activity, then make the beta catenin dephosphorylate in the steady state. The beta catenin is accumulated and activates LEF/Tcf to promote transcription, which can enable the expression of AP-1 and prompt expression of CyclinD1 and MMP-9 expression and so on. The changes could result in proliferation, invasion and metastasis of tumor cells [33–35]. Meanwhile, we also detected some crucial target molecules to assess whether that RI regulates ILK signaling pathways is truly very efficient. The results revealed that RI suppressed the expression of the ILK and phosphorylation of PKB/Akt, GSK-3β and other important molecules in this signaling pathway. Moreover, the results indicated that PTEN expressions were significantly increased in EJ-RI cells, but phosphorylated PTEN was up-regulating in EJ-ILK cells groups, compared with control group cells respectively. PTEN was activated due to decrease of phosphorylated PTEN level, and then inhibited downstream AKT activation. The role of PTEN is serving on a tumor suppressor gene by suppressing Akt/PKB signaling pathway. The activity of ILK is suppressed by phosphatases such as ILKAP and PTEN [36], our results agree with this research.

To gain invasive capabilities, tumor cells go through a morphology and physiology change such as EMT. The invasion of cancer cells is remarkably affected by EMT [33]. Firstly, we showed that up-regulating ILK leads to the cell phenotype from epithelial to mesenchymal morphological changes. Secondly, in this study, we showed that ILK increase caused a decrease of E-cadherin as epithelial cell marker and an ascent of Vimentin and N-cadherin as mesenchymal markers, and enhanced the expression of Snail and Twist as EMT inducer. The most obvious change of EMT is the down-regulating E-cadherin and the acquisition the expression of EMT regulators such as Snail, Slug and Twist [37]. Thirdly, We also showed that over-expression of ILK could increase the protein level of MMP-2 and MMP-9. It has been reported MMPs have something to do with EMT in cancer progression [38]. our experiment results supported the view that ILK is an important regulator of EMT [39]. Conversely, we demonstrated that RI affected the expressions of these EMT-related proteins in EJ cells, the results showed that over-expression of RI remarkably restrained EMT of EJ cells, which is in line with previous report [40].

Finally, the animal model experiment demonstrated that ILK significantly promoted growth and metastasis of transplanted tumor; tumors of EJ-ILK cell group were heavier and the microvascular density is higher than those in the control groups. Direct down-regulation of ILK caused a reduction of endothelial cell growth and blood vessel formation in vivo [41]. Moreover, fewer metastases of the lung were detected in the EJ-RI group; and that, EJ-ILK cell groups showed a remarkable invasion and metastases. In particular, we found the expression of S100A4 markedly enhanced in EJ-ILK cells group compared other groups in vivo and vitro. S100A4 secreted by tumor and stromal cells, is a member of the S100 calcium-binding protein family. It can support tumorigenesis by triggering angiogenesis. Recently, S100A4 has attracted the attention of researchers because its expression is related to the movement, invasion, metastasis, angiogenesis and prognosis of various tumors [42], which agree well with our findings. Our study also proved that RI inhibited ILK expression and suppressed angiogenesis both in vivo and in vitro. Interestingly, our observation on the human pathological specimens consistently revealed that RI and ILK negatively correlated in tumor and non-tumor tissues, which is corresponding to in vitro and animal experiments.

## Conclusions

Taken together, for the first time, our data demonstrated that RI might regulate the function of ILK through mutual binding. Therefore, we propose that RI could suppress tumorigenesis, EMT and metastasis by regulating ILK mediated signal pathway. The data provided evidence that ILK could be a valuable therapeutic and diagnostic target for bladder cancer. Our research also highlights the possibilities that RI could serve as a promising anti tumor protein and angiogenesis inhibitor. Further research will be required to verify the underlying molecular mechanism of the interaction between ILK and RI.
